# Androgen receptor expression is required to ensure development of adult Leydig cells and to prevent development of steroidogenic cells with adrenal characteristics in the mouse testis

**DOI:** 10.1186/s12861-019-0189-5

**Published:** 2019-04-17

**Authors:** Peter J. O’Shaughnessy, Rod T. Mitchell, Ana Monteiro, Laura O’Hara, Lyndsey Cruickshanks, Hedi Claahsen-van der Grinten, Pamela Brown, Margaret Abel, Lee B. Smith

**Affiliations:** 1 College of Medical, Veterinary and Life Sciences, Institute of Biodiversity, Animal Health and Comparative Medicine, University of Glasgow, G61 1QH, Glasgow, UK; 2 MRC Centre for Reproductive Health, University of Edinburgh, The Queen’s Medical Research Institute, 47 Little France Crescent, Edinburgh, EH16 4TJ UK; 3 Centre for Discovery Brain Sciences, University of Edinburgh, Hugh Robson Building, George Square, Edinburgh, EH8 9XD UK; 4 Department of Paediatrics, Radboud Amalia Children’s Hospital, Radboud University Medical Center, Nijmegen, The Netherlands; 5 Department of Human Anatomy and Genetics, University of Oxford, South Parks Rd, Oxford, OX1 3QX UK; 6 School of Environmental and Life Sciences, University of Newcastle, Callaghan, NSW 2308 Australia

**Keywords:** Testis, Androgen receptor, Leydig, Adrenal, Development, Stereology

## Abstract

**Background:**

The interstitium of the mouse testis contains Leydig cells and a small number of steroidogenic cells with adrenal characteristics which may be derived from the fetal adrenal during development or may be a normal subset of the developing fetal Leydig cells. Currently it is not known what regulates development and/or proliferation of this sub-population of steroidogenic cells in the mouse testis. Androgen receptors (AR) are essential for normal testicular function and in this study we have examined the role of the AR in regulating interstitial cell development.

**Results:**

Using a mouse model which lacks gonadotropins and AR (*hpg*.ARKO), stimulation of luteinising hormone receptors in vivo with human chorionic gonadotropin (hCG) caused a marked increase in adrenal cell transcripts/protein in a group of testicular interstitial cells. hCG also induced testicular transcripts associated with basic steroidogenic function in these mice but had no effect on adult Leydig cell-specific transcript levels. In *hpg* mice with functional AR, treatment with hCG induced Leydig cell-specific function and had no effect on adrenal transcript levels. Examination of mice with cell-specific AR deletion and knockdown of AR in a mouse Leydig cell line suggests that AR in the Leydig cells are likely to regulate these effects.

**Conclusions:**

This study shows that in the mouse the androgen receptor is required both to prevent development of testicular cells with adrenal characteristics and to ensure development of an adult Leydig cell phenotype.

**Electronic supplementary material:**

The online version of this article (10.1186/s12861-019-0189-5) contains supplementary material, which is available to authorized users.

## Background

The two major steroidogenic cell types in the male are the adrenocortical cell and the testicular Leydig cell. Both cell types share a basic steroidogenic cell phenotype typified by expression of proteins such as CYP11A1, STAR and HSD3B with added expression of cell-specific enzymes such as CYP11B1 (required for corticosterone production in adrenocortical cells) or HSD17B3 (required for testosterone production in adult Leydig cells). Embryonic stem cells, mesenchymal cells or fibroblasts can be induced towards a steroidogenic lineage [1–3] and the transformed cells will follow an adrenocortical or Leydig cell lineage or take on the phenotype of the more basic steroidogenic cell [1, 4, 5]. Leydig cells and adrenocortical cells are closely linked, therefore, and there is evidence that the two cell types originate from a single cell population [6]. While many of the factors involved in steroid cell differentiation are known [7], it is not clear what controls the final phenotype that the cells will adopt, although the local environment is likely to make a major contribution.

During fetal development the adrenals and testes form from a single adreno-gonadal primordium [6] which functionally divides in the mouse at about embryonic day (e) 11.5 [8]. Following adreno-gonadal separation the testes have been shown to contain a number of cells with adrenal characteristics and there is evidence that these may be derived from a cohort of adrenal cells that migrates to the developing gonad and remains there after adrenal/gonadal separation [9]. Shortly after the split of the adreno-gonadal primordium the fetal population of Leydig cells develops in the testicular interstitium and so it is also possible that some or all of the adrenal-like cells may be a normal sub-population of the developing Leydig cells [10–12]. After birth, between days 7 and 10 in the mouse [13, 14], a second post-natal or “adult” population of Leydig cells starts to differentiate from progenitor cells lying mainly in the peritubular region [15–18]. During development and in the adult, therefore, current evidence suggests that the testicular interstitium normally contains fetal and adult Leydig cells and a small number of cells with adrenal characteristics which are of uncertain origin [9, 19]. Development of the adult Leydig cell population is very largely dependent upon the action of luteinising hormone (LH) but it is not clear why the testicular adrenal cells do not show further development since they also respond to LH [9].

Using a number of different models of androgen receptor (AR) ablation, previous studies have shown that AR are required for normal Leydig cell development in the adult [19–23]. In testicular feminised (*Tfm*) mice, which lack AR due to a mutation in the gene, and in ARKO mice adult Leydig cell number is reduced by up to 50% [20, 22] and the profile of steroidogenic enzyme expression is markedly altered [20]. Differences in enzyme expression may be complex as LH levels are increased in ARKO and *Tfm* mice [22, 24] but the results suggest that both LH and the AR may interact to ensure that there is normal proliferation and differentiation of testicular steroidogenic cells and that these cells adopt a specific Leydig cell phenotype.

To examine the role of LH and androgen in regulating development of interstitial steroidogenic cells (both Leydig cells and cells with adrenal characteristics) we have used the hypogonadal (*hpg*) mouse crossed with animals lacking ARs. Three models have been used; the *hpg* mouse which lacks circulating gonadotrophins [25] and is responsive to both LH and androgens, the *hpg*.SCARKO mouse which lacks AR specifically on the Sertoli cells and the *hpg*.ARKO mouse which lacks AR ubiquitously. The testicular phenotype of these animals has been described previously [26] and they differ from SCARKO, ARKO and *Tfm* models in that they lack gonadotrophins. This means that the Leydig cells in all animals will be largely inactive and under-developed but they will also be highly sensitive to the effects of exogenous hormone stimulation [27–29].

Results from this study show that the AR is essential for both LH-induced development of the adult Leydig cell phenotype and to prevent development of cells with adrenal characteristics in the testicular interstitium through probable action within the Leydig cells.

## Results

### hCG-induced Leydig cell hyperplasia in the hpg mouse is dependent on androgen receptors

**Fig. 1:**
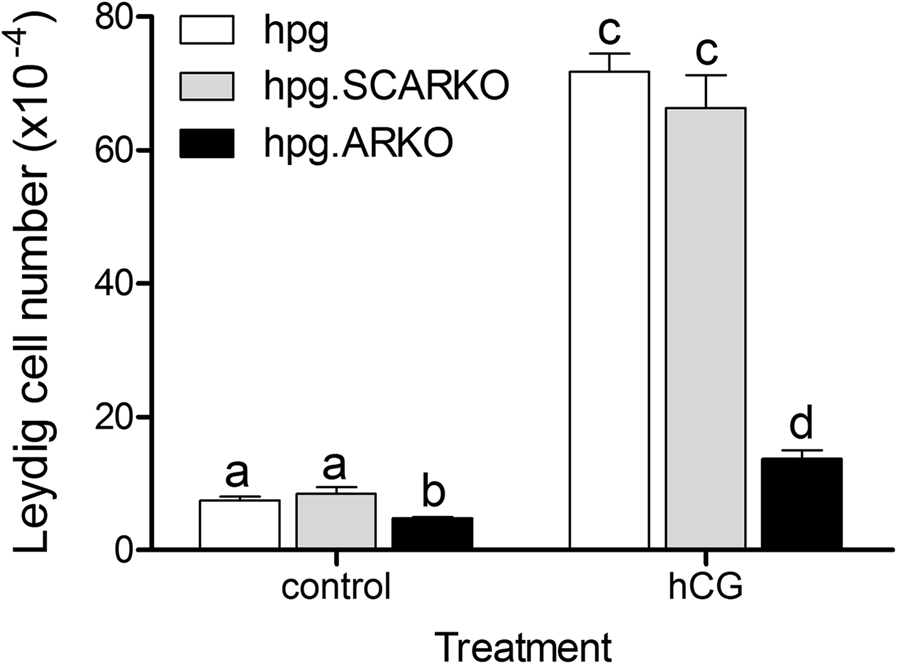
Treatment with hCG increases Leydig cell number in *hpg*, *hpg*.SCARKO and *hpg*.ARKO mice. Adult mice were treated daily with 4 IU of hCG (or vehicle) for 1 week and Leydig cell number counted using the optical disector method. Results show the mean ± SEM of between 3 and 5 animals per group. Groups with different letter superscripts are significantly (P < 0.05) different. Primary raw data are shown in Additional file 1

Treatment with human chorionic gonadotropin (hCG; homologous protein to LH that acts on the LH-receptor) increased testicular volume (Table 1) and caused an 8 to10-fold increase in total Leydig cell number (Fig. 1) in both *hpg* and *hpg*.SCARKO mice compared to untreated animals. In *hpg*.ARKO mice, treatment with hCG had no effect on testicular volume and increased Leydig cell number by 3-fold, indicating a requirement for androgen action to achieve the full hyperplastic effect of hCG (Fig. 1). All data shown as mean ± sem in Fig. 1 (and subsequent figures) are also shown as primary raw data in Additional file 1.

**Table 1:** Effect of hCG on testicular volume

	Testicular volume (mm^3^)	
genotype	control	plus hCG
*hpg*	1.80 ± 0.16	6.12 ± 0.73^*^
*hpg*.SCARKO	1.70 ± 0.14	4.38 ± 0.12^*^
*hpg*.ARKO	0.83 ± 0.08	1.30 ± 0.06

*Significantly (*P* < 0.05) different to control by ANOVA and t-test

### Expression of transcript/proteins common to most steroidogenic cells is unaffected by the absence of androgen receptors

**Fig. 2:**
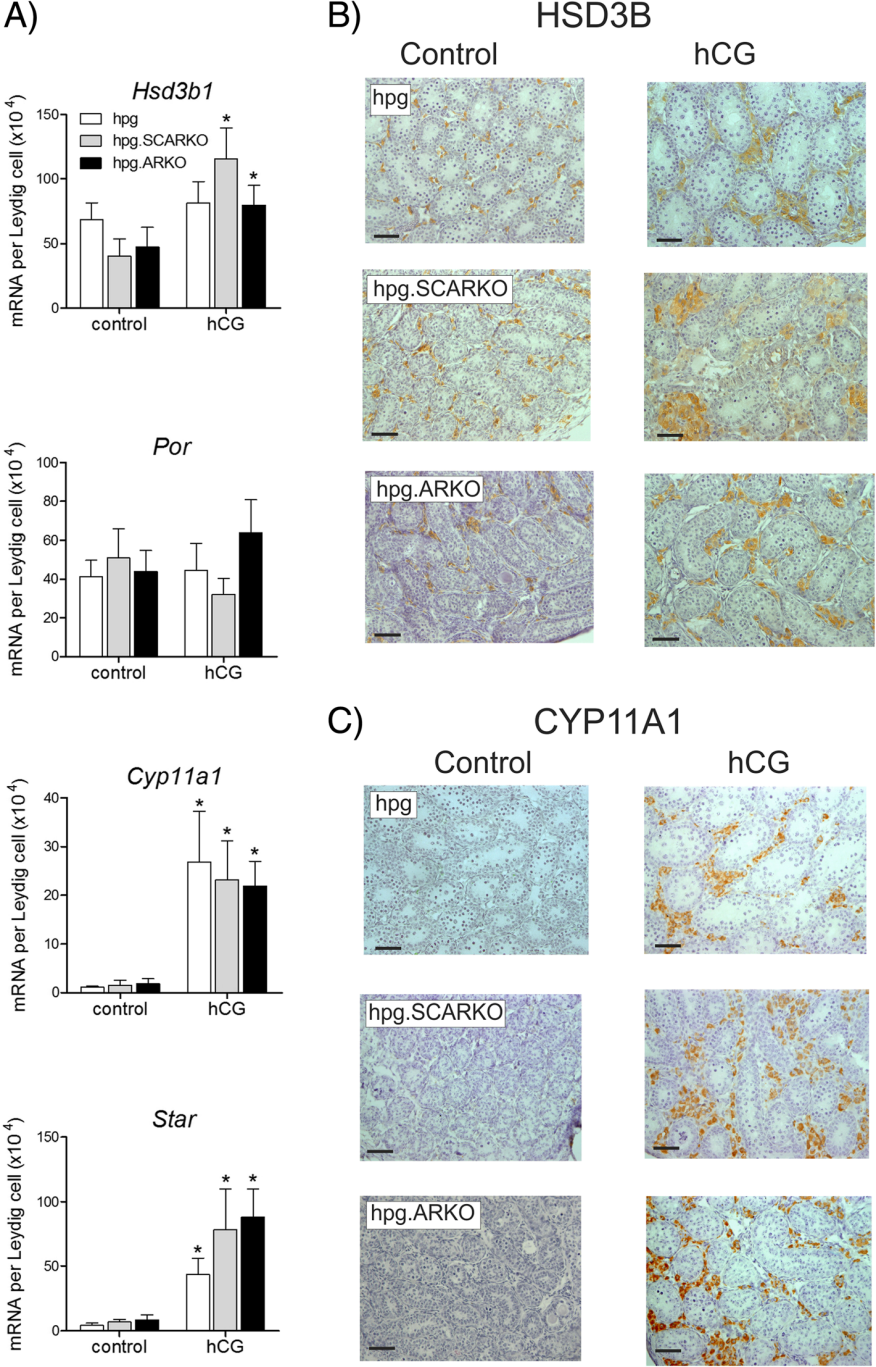
hCG-induced expression of transcript/proteins common to most steroidogenic cells is unaffected by the absence of androgen receptors. Adult *hpg*, *hpg*.SCARKO and *hpg*.ARKO mice were treated daily with 4 IU of hCG (or vehicle) for 1 week. **a** Expression of *Hsd3b1*, *Por*, *Cyp11a1* and *Star* was measured by qPCR and is expressed relative to Leydig cell number in each group. The presence of an asterisk (*) indicates that the effect of hCG was significant (P < 0.05) for that mouse group relative to the respective control. **b** and **c** Immunohistochemical expression of HSD3B and CYP11A1 in testes from *hpg*, *hpg*.SCARKO and *hpg*.ARKO mice with or without hCG treatment. The bar represent 50 μm. Primary raw data are shown in Additional file 1

Expression of *Hsd3b1* and *Por* transcripts was relatively high in untreated *hpg*, *hpg*.SCARKO and *hpg*.ARKO mice and showed little response to hormone treatment (Fig. 2a). This was also reflected in the clear immunohistochemical staining for HSD3B (Fig. 2b) seen in all groups with or without hCG treatment. The main effect of hCG was to increase the number of cells expressing HSD3B, consistent with the total increase in Leydig cell numbers induced by hCG, although there was some variation in staining intensity. In contrast to *Hsd3b1*, *Cyp11a1* and *Star* transcript levels were very low in untreated mice but were clearly stimulated by hCG in all three groups (Fig. 2a). Similarly, CYP11A1 was largely undetectable by immunohistochemistry in untreated animals from any group but showed marked interstitial expression in all groups following hCG (Fig. 2c).

### hCG induces adult Leydig cell-specific transcripts only in the presence of androgen receptors

**Fig. 3:**
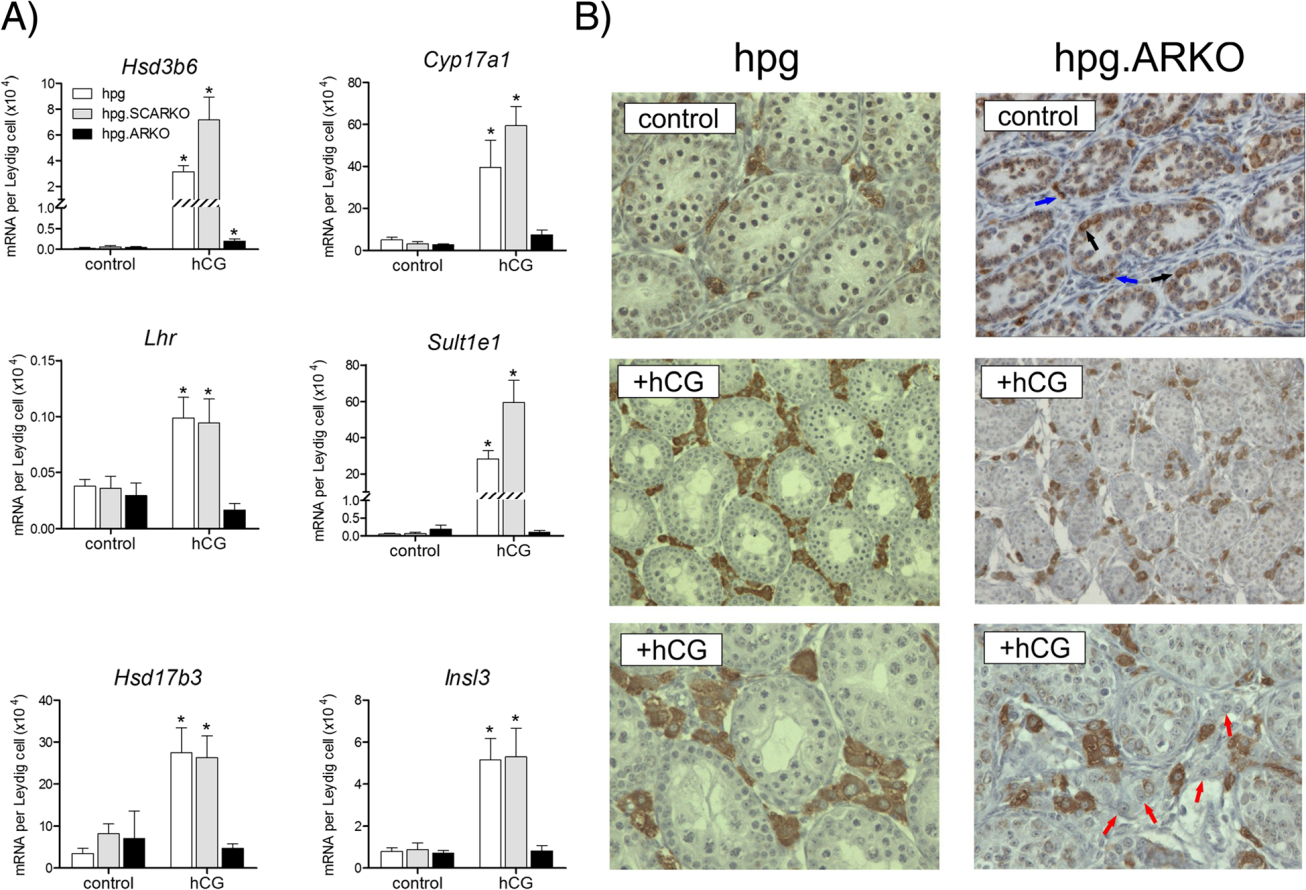
Treatment with hCG induces adult Leydig cell-specific transcripts only in the presence of androgen receptors. Adult in *hpg*, *hpg*.SCARKO and *hpg*.ARKO mice were treated daily with 4 IU of hCG (or vehicle) for 1 week. **a** Expression of *Hsd3b6*, *Cyp17a1*, *Lhr, Sult1e1, Hsd17b3* and *Insl3* was measured by qPCR and is expressed relative to Leydig cell number in each group. The presence of an asterisk (*) indicates that the effect of hCG was significant (P < 0.05) relative to control for that mouse group. **b** Immunohistochemical expression of CYP17A1 in testes from *hpg* and *hpg*.ARKO mice with or without hCG treatment . In control *hpg*.ARKO mice the blue arrows identify interstitial cells which express CYP17A1 while the black arrows identify germ cells which express the enzyme. The red arrows indicate a number of likely Leydig cells which do not express CYP17A1 following hCG treatment. Primary raw data are shown in Additional file 1

Transcript levels of *Cyp17a1*, *Lhr*, *Hsd17b3*, *Insl3*, *Sult1e1* and *Hsd3b6*, which are all Leydig cell specific [13, 30–32], were significantly increased by treatment with hCG in *hpg* and *hpg*.SCARKO mice. In contrast, there was little or no effect of hCG on transcript levels in *hpg*.ARKO mice (Fig. 3a). Immunohistochemical staining for CYP17A1 showed that the protein was detectable in untreated *hpg*, and *hpg*.ARKO mice (Fig. 3b) despite low transcript levels. Following hCG treatment there was a clear increase in the number of cells expressing CYP17A1 in the *hpg* mice. In *hpg*.ARKO mice there was also an increase in interstitial cells expressing CYP17A1 following hCG treatment, consistent with the increase in Leydig cell number, but a large number of unstained cells with a Leydig cell morphology were also present in the interstitium (Fig. 3b, red arrows). Interestingly, in the untreated *hpg*.ARKO mice there was clear immunostaining of the spermatogonia (Fig. 3b, black arrows). This staining was not seen in *hpg* mice (Fig. 3b) or in *hpg*.SCARKO mice (not shown) and was largely undetectable in the *hpg*.ARKO after hCG-treatment.

### hCG induces adrenal transcripts/protein in a number of interstitial cells in the absence of androgen receptors

**Fig. 4:**
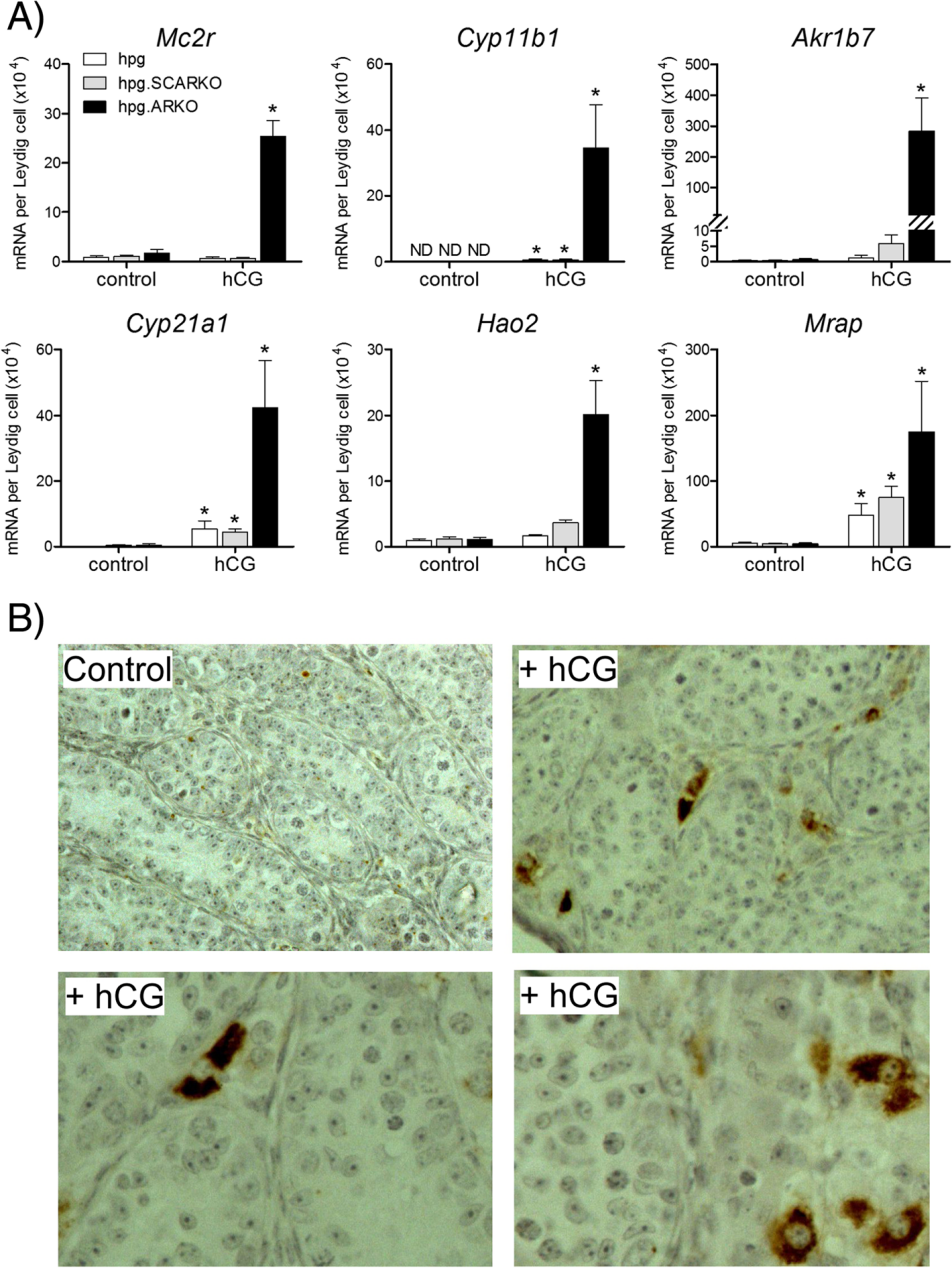
hCG-treatment induces adrenal transcripts/protein in the absence of androgen receptors. Adult *hpg*, *hpg*.SCARKO and *hpg*.ARKO mice were treated daily with 4 IU of hCG (or vehicle) for 1 week. **a** Expression of *Mc2r*, *Cyp11b1*, *Akr1b7, Cyp21a1, Hao2* and *Mrap* was measured by qPCR and is expressed relative to Leydig cell number in each group. The presence of an asterisk (*) indicates that the effect of hCG was significant (P < 0.05) for that mouse group relative to control. **b** Immunohistochemical expression of CYP11B1 in testes from *hpg*.ARKO mice with or without treatment with hCG. Primary raw data are shown in Additional file 1

Expression of transcripts associated with adrenocortical steroidogenic cells (*Mc2r*, *Cyp11b1*, *Cyp21a1*, *Akr1b7*, *Mrap* and *Hao2*) showed marked stimulation in response to hCG in *hpg*.ARKO mice (Fig. 4a). In *hpg* and *hpg*.SCARKO mice, in contrast, hCG either had no effect on these transcripts or caused only a small increase in expression (Fig. 4a). Consistent with transcript data, CYP11B1 was undetectable by immunohistochemistry in control or hCG-treated *hpg* or *hpg*.SCARKO mice (not shown). In *hpg*.ARKO mice occasional interstitial cells stained for CYP11B1 but most were negative. Treatment with hCG, however, led to more intense staining although the numbers of steroidogenic cells showing expression remained low (Fig. 4b).

### AR expression in the Leydig cells inhibits development/differentiation of cells with adrenal characteristics in the testis

**Fig. 5:**
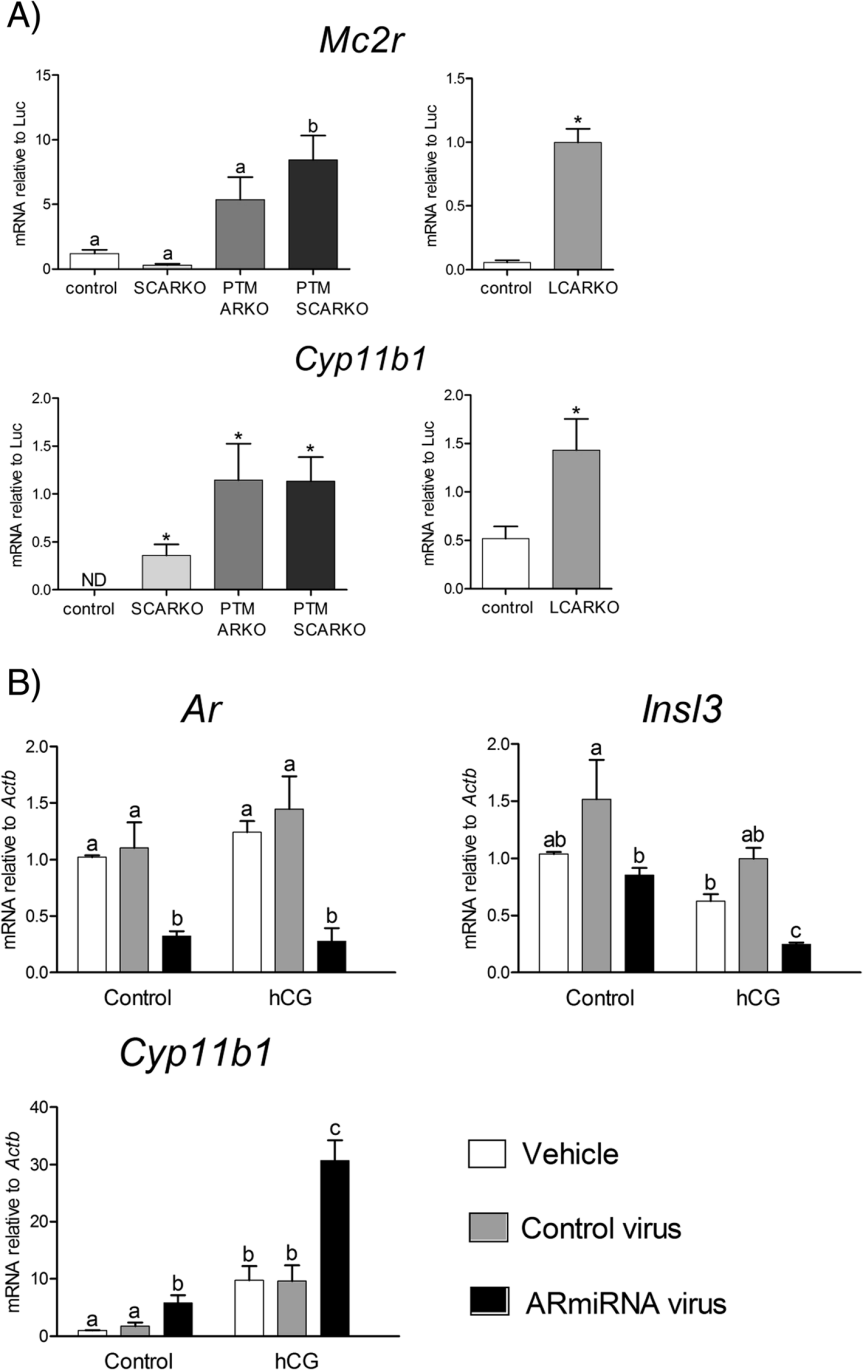
Control of adrenal cell development/differentiation in the testis depends largely on AR expressed in the Leydig cells and peritubular myoid cells. **a** Testicular expression of *Mc2r* and *Cyp11b1* in mice lacking ARs in different cell types. Transcript levels are measured relative to the external standard (*Luc*). Data were analysed by single-factor ANOVA or t-test, apart from *Cyp11b1* levels in SCARKO, PTM.ARKO, PTM.SCARKO mice which was analysed initially using the Kruskal-Wallis test. The Kruskal-Wallis test showed a significant (*P* < 0.001) overall difference between groups which were further analysed using single sample t-tests which showed each group was significantly different to control. Different letter superscripts indicate differences (P < 0.05) between groups (analysed by ANOVA) while the presence of an asterisk indicates a significant difference to control (analysed by t-test). For all data, the mean ± SEM of between 7 and 9 animals in each group is shown. **b** Effect of androgen receptor (AR) knockdown on transcript levels in a Leydig tumor cell (MLTC-1) line. Cells were transfected with vehicle, lentivirus containing control, “scrambled” miRNA or lentivirus containing miRNAs which target the *Ar*. Data was analysed by 2-factor ANOVA and groups with different letter superscripts are significantly different. Each group shows the mean ± SEM of 3 or 4 individual experiments. Primary raw data are shown in Additional file 1

Data above show that preventing differentiation of cells with adrenal characteristics in the testis depends on AR expression. To determine the cell types in which AR expression is essential for this effect we examined testicular adrenal transcript levels in different cell-specific models of AR deletion. Data in Fig. 5a shows that the Sertoli cell AR are unlikely to be the major factor responsible for the effects seen as there was no change in *Mc2r* in the SCARKO mouse, although there was an increase in *Cyp11b1* transcript levels in the same animals. In contrast, there were more marked changes in both *Mc2r* and *Cyp11b1* in mouse models lacking AR in the PTM cells (Fig. 5a). It is likely, however, that PTM.ARKO and PTM.SCARKO mice also lack AR in at least some of the Leydig cells as PTM cells are reported to act as Leydig cell precursors [23, 33]. To test this we examined *Mc2r* and *Cyp11b1* transcript levels in Leydig cell-ARKO (LCARKO) mice (which are on a different background to the other mouse models [23, 34, 35]) (Fig. 5a). Results show that both *Mc2r* and *Cyp11b1* are significantly increased in testes specifically lacking AR from Leydig cells, suggesting that the site of action of AR necessary for Leydig cell specification (as opposed to adrenal-like specification) is primarily the Leydig cells themselves.

To examine, directly, the effect of AR depletion in mouse Leydig cells we used RNAi to knockdown AR in a mouse Leydig tumor cell (MLTC-1) line. Exposure of MLTC-1 cells to AR miRNA significantly reduced *Ar* transcript levels with or without hCG treatment (Fig. 5b) (mean 67.5% knockdown). A reduction of *Ar* in these cells was associated with a significant reduction in transcript levels of the LC-specific androgen target gene *Insl3* [36]. Suppression of AR also resulted in a significant increase in the adrenal enzyme *Cyp11b1* (Fig. 5b).

## Discussion

During fetal development the gonads and adrenals initially form adjacent to each other from the same priomordium and there is evidence that the same SF1-positive cells give rise to adrenocortical cells and fetal Leydig cells [6, 37]. Subsequently, the adrenals and gonads separate, at about embryonic day 11 in the mouse, and some cells from the adrenal may become assimilated into the developing gonad [9] despite normal inhibition of the process by WNT4 [8]. Under this scenario, these cells would give rise to the adrenal-like cells seen in the adult testis. Cells with adrenal characteristics may also arise as a small subgroup of the developing Leydig cells [10, 11] and it is possible that there may be more than one such population of cells in the testicular interstitium. Whatever their origin, these adrenal-like cells in the mouse testis remain quiescent postnatally and it is only in humans that they have been shown to proliferate and to become steroidogenically active if stimulated by high ACTH [38]. Results described here now suggest the quiescent nature of these cells in mice is dependent upon testicular ARs which act to inhibit inappropriate, LH-dependent, postnatal development of cells with adrenal characteristics. Results also show that, in the absence of ARs, trophic stimulation of steroidogenic cells in the testicular interstitium leads to development of a basic steroidogenic cell type lacking enzymes specific for the adult Leydig cells.

Data from the *hpg* mouse, and other models of gonadotrophin or LH deficiency, have shown previously that LH is required for the establishment of a normal population of active Leydig cells. In the absence of LH or the LH-receptor Leydig cell number is markedly reduced [39–41] and Leydig cell activity is minimal [40, 41] while treatment with LH/hCG increases both cell number and activity [27, 42, 43]. Earlier studies have suggested that the adult *hpg* testis contains adult-type Leydig cells [44] and that the main effect of LH treatment is likely to be proliferation and maturation of these cells rather than induced stem cell differentiation [44]. This is consistent with other studies which have shown increased [^3^H]thymidine labelling of Leydig cells in the *hpg* mouse following LH treatment [27]. In addition, it is clear from the pattern of HSD3B and CYP11A1 staining after hCG treatment, in the current study, that there is an overall increase in Leydig cell numbers throughout the interstitium. This contrasts with the marked increase in peritubular Leydig cells that occurs during Leydig cell stem cell differentiation following ethanedimethane sulphonate (EDS) treatment [15, 45, 46].

All steroidogenic cells express a base-line level of the proteins CYP11A1 and STAR which are required to form C21 steroids from cholesterol and POR is expressed if there is further metabolism by P450 enzymes in the endoplasmic reticulum. HSD3B is also generally expressed in steroidogenic cells although it is lacking in some cases (e.g. most human fetal adrenal cells [47, 48]). The different steroidogenic cell types are distinguished largely through the additional enzymes and hormone receptors that they express. In this study, the common steroidogenic transcripts were either expressed constitutively in the interstitial steroidogenic cells of the *hpg* or were induced by LH/hCG but, in either case, there was no influence from the presence or absence of ARs. This would suggest that LH/hCG acts to induce generic steroidogenic potential in the interstitial steroidogenic cells of the testis and that additional stimulation of the ARs is essential for the induction of a Leydig cell phenotype, consistent with previous data from androgen-resistant *Tfm* and LCARKO mice [20, 23]. Both gonadal and adrenal steroidogenic cells are dependent on SF-1 which regulates downstream expression of genes required for steroiodogenesis [49]. It has been shown that forced expression of SF-1 in stem cells will transform those cells into steroidogenic cells [4]. The phenotype that the transformed cells adopt is variable and depends on a combination of stem cell type, SF-1 dosage and other transcriptional factors [2–4] but, in vivo, other factors are likely to determine the tissue-specific cell type. The results reported here now suggest that the AR is a required factor for generation of the adult Leydig cell phenotype in vivo. An alternative explanation would be that the *hpg*.ARKO mouse contains only fetal-type Leydig cells and LH/hCG induces proliferation and stimulates activity in these cells [50]. This would certainly be consistent with the failure of adult Leydig cell markers to increase after hCG. Recent studies have shown, however, that while there is a relative increase in fetal Leydig cells in the adult ARKO mouse, adult Leydig cells still outnumber fetal Leydig cells by a factor of about 4 to 1 [19]. Thus, lack of ARs does not appear to prevent at least initial differentiation and development of the adult Leydig cell population, consistent with previous results [23].

It appears likely that the cells showing induced adrenal transcript expression in the *hpg*.ARKO mice after hCG injection are the cells with adrenal characteristics cells described earlier [9]. This would be consistent with the relatively small number of cells in the *hpg*.ARKO testes that show an increase in adrenal enzymes in response to hCG treatment. Overall, the failure of hCG to induce adrenal transcript expression in *hpg* mice indicates that ARs normally inhibit trophic hormone stimulation of cells with adrenal characteristics in the testis. Interestingly, and consistent with this data, recent studies have shown that androgens act to inhibit recruitment and differentiation of progenitor cells into steroidogenic cells in the mouse adrenal [51]. Relevant data has also emerged from the study of *Gata4* and *Gata6* double knockout mice. In these animals there is loss of Leydig cells [52, 53] but survival and, perhaps, proliferation of cells with adrenal characteristics in the testis [52]. This indicates that there may be some synergism between the ARs and GATA factors in these interstitial cells, as seen in other tissues [54].

Androgen receptors are expressed in the Sertoli cells, the PTMC, vascular smooth muscle, endothelial cells and the Leydig cells in the normal post-natal mouse testis [26, 55] and a similar pattern of expression is seen in *hpg* mice [26]. Since the effects of hormone stimulation are similar in *hpg* and *hpg*.SCARKO mice, the results from this study show that androgen must mediate its effects on Leydig/adrenal cell development either through the cells themselves or through the PTMC, but not through the Sertoli cells. Previous studies using the Leydig cell specific LCARKO mouse have shown that it recapitulates many of the effects on Leydig cell transcript expression seen in the ARKO mouse [23] and we show here that there is increased adrenal transcript expression in LCARKO mice, and also when AR is specifically knocked down in vitro in a Leydig cell line. This suggests that the main effects of androgen on Leydig cell development are mediated through ARs in the Leydig cells. As the origin of testicular cells with adrenal characteristics is uncertain, however, it is not clear whether ARs expressed in these cells or the Leydig cells regulates their development in the testis. Nevertheless, data from AR knockdown in MLTC-1 cells suggests that there may be a requirement for continued AR-mediated suppression of plasticity, since a reduction of AR in MLTC-1 cells is able to switch ‘mature’ Leydig cells towards an adrenal cell fate.

Recent work has suggested that *Hsd3b1* in the mouse testis is only expressed in fetal Leydig cells [56] which would suggest that the adult *hpg* mouse testis contains a significant number of fetal Leydig cells. This is consistent with the “independent” nature of these cells which do not require LH stimulation during development [57, 58] or input from the Sertoli cells [59]. Expression of *Hsd3b1* was also relatively unaffected by the presence or absence of ARs indicating that ARs are not important for fetal Leydig cell development, as previously suggested [19].

These studies underline the importance that the ARs play in regulating development and function of cells in the testicular interstitium. This data, and previous studies [44], show that the interstitial tissue of the adult *hpg* mouse testis contains adult Leydig cells and cells with adrenal characteristics and that hCG/LH treatment stimulates Leydig cell proliferation/activity while having little effect on the other cells. The *hpg*.ARKO mouse also contains adult Leydig cells and cells with adrenal characteristics but appears to have an additional population of “basal” steroidogenic cells. In these animals, hCG treatment causes proliferation of the adult Leydig cells but has little effect on adult Leydig cell-specific transcripts (Fig. 3). hCG-treatment also causes the basal steroidogenic cells to undergo proliferation and stimulation (Figs. 2 and 3) and leads to activation of adrenal-like cells (Fig. 4).

## Conclusions

These studies have dissected the roles that LH (hCG) and the AR play in the development of interstitial cells in the mouse testis. As shown before, LH/hCG stimulates proliferation and maturation of Leydig cells but we show here that this process requires the presence of AR in the Leydig cells. These AR are also required to prevent activation of cells with adrenal characteristics under LH/hCG stimulation and act, therefore, to induce the formation of normal interstitial cell populations in the adult mouse testis.

## Methods

### Animals and treatments

ARKO, SCARKO, *hpg*.ARKO and *hpg*.SCARKO mice were generated at the University of Oxford as published previously [﻿1﻿6] from mice obtained originally from Prof G Verhoeven and Dr. K De Gendt (Catholic University of Leuven, Belgium) and from mouse colonies bred at the University of Oxford [16]. SCARKO and ARKO mice were produced by crossing female mice carrying an *Ar* with a floxed exon 2 (Ar^fl^) with male mice expressing Cre under the regulation of the Sertoli cell-specific promoter *Amh* or the ubiquitous promoter *Pgk-1* [60–62]. LC-ARKO mice were generated at the University of Edinburgh by mating Ar^fl^ female mice (obtained as above) with male mice expressing Cre under the regulation of the *Fabp4* promoter (provided by Kerry McInnes, University of Edinburgh) [23] while PTM-ARKO were generated as previously described [34] by mating Ar^fl^ female mice with male mice expressing Cre under the regulation of the *Myh11* promoter (originally from Michael Kotlikoff (Cornell University, USA)) [35]. Mice were killed, as necessary, by exposure to a rising concentration of CO_2_.

Adult mice (10 weeks) were injected sub-cutaneously with 4 IU recombinant hCG (Serono Ltd. Middlesex, UK) in 0.2 ml PBS (phosphate buffered saline, pH 7.4, Sigma Aldrich, Dorset, UK) once daily for 7 days. hCG acts as an LH analog and stimulates Leydig cell function through the LH-receptor. Mice were killed, as above, on day 8 (24 h after the last injection) and testes snap frozen in liquid N_2_ or fixed in Bouin’s overnight.

### Stereology

For stereological analysis, testes were embedded in Technovit 7100 resin, cut into sections (20 μm), and stained with Harris’ hematoxylin. The total testis volume was estimated using the Cavalieri principle [63]. The optical disector technique [64] was used to count the number of Leydig cells in each testis. Leydig cells were recognised by their position, round nucleus and relatively abundant cytoplasm [39, 65]. The numerical density of each cell type was estimated using an Olympus BX50 microscope fitted with a motorized stage (Prior Scientific Instruments, Cambridge, UK) and Stereologer software (Systems Planning Analysis, Alexandria, VA, USA). Stereological data for untreated *hpg*, *hpg*.SCARKO and *hpg*.ARKO animals in this study includes data from a previous study [62] plus additional animals. Testicular volume data (Table 1) also includes data reported previously [26, 62] with the inclusion of additional samples.

### Immunohistochemistry

For immunohistochemistry, testes were embedded in paraffin and sections (5 μm) were mounted on glass slides, dewaxed, and rehydrated. Sections were incubated with anti-CYP11A1 (rabbit polyclonal, donated by Dr. AH Payne, Stanford University), anti-HSD3B2 (rabbit polyclonal, donated by Prof Ian Mason, University of Edinburgh), anti-CYP11B1 (mouse polyclonal, donated by Prof Celso E. Gomez-Sanchez, University of Mississippi) after heat-induced antigen retrieval. Bound primary antibody was detected using a peroxidase-conjugated secondary antibody, followed by a fluorescyl-tyramide amplification step with visualization using 3,3-diaminobenzidine tetrahydrochloride (Dako UK Ltd., Cambridgeshire, UK). To reduce “mouse-on-mouse” effects when using an anti-mouse second antibody blocking reagent (M.O.M. Vector Laboratories Ltd., Peterborough, UK) was used. For negative control samples, non-immune serum replaced primary antiserum.

### Cell culture and RNAi

MLTC-1 cells (mouse Leydig cell tumor, ATCC® Number: CRL-2065™) were maintained in culture at 37 °C with 5% CO_2_ in 89% RPMI-1640 media (containing 2.5 mM L-Glutamine, 0.5 mM Sodium pyruvate, 1.2 g/L Sodium bicarbonate and 15 mM HEPES), 10% fetal bovine serum and 1% penicillin/streptomycin. Cells were transfected with either an AR miRNA lentivirus consisting of a CMV promoter, an mKate2 fluorescent reporter and 2 chained miRNAs that target the androgen receptor (“Lc-cppt-CMV-mKate2-ARmiR2&4”), a ‘scrambled’ lentivirus (“Lv-cppt-CMV-mKate2-negmiRx2”) or a control with no virus. Viruses were produced by Dr. Pamela Brown at the SURF University of Edinburgh Biomolecular Core Facility. Transfection was performed at an MOI of 5 with medium supplemented with a final concentration of 6 μg/mL Polybrene (Sigma). Cells were then sorted by Fluorescence Activated Cell Sorting for mKate2 expression to increase the purity of the transfected cell population before being selected for blasticidin resistance. To treat cells with hCG, cells growing in complete medium were allowed to reach confluence, then replaced with serum-free medium and incubated for four hours. The serum-free medium was then removed and replaced with either serum-free medium supplemented with 1.5 IU hCG final concentration for “+hCG” groups or serum-free medium only for “-hCG” groups. The cells were then incubated for 16 h before being collected and processed for RNA extraction. Each condition was repeated four times with two or three flasks each time used as technical replicates.

### Measurement of mRNA levels

For data shown in Figs. 1, 2, 3 and 4, testis RNA was extracted using TRIzol (Life Technologies, Paisley, UK) and the RNA was reverse transcribed using random hexamers and Moloney murine leukemia virus reverse transcriptase (Superscript III, Life Technologies, Paisley, UK) as described previously [66]. To allow specific mRNA levels to be expressed per testis and to control for the efficiency of RNA extraction, RNA degradation, and the reverse transcription step, an external mRNA standard was used [67, 68]. The external standard was luciferase mRNA (Promega UK, Southampton, UK), and 1 ng was added to each testis at the start of the RNA extraction procedure. To measure transcript levels, real-time PCR was used with the SYBR green method in a 96-well plate format using an MX3000 cycler (Agilent Technologies, Berkshire, UK). Reactions contained 5 μl 2 x SYBR mastermix (Agilent Technologies, Berkshire, UK), primer (100 nM) and template in a total volume of 10 μl. At the end of the amplification phase a melting curve analysis was carried out on the products formed. All primers were designed by Primer Express 2.0 (Applied Biosystems, Warrington, UK) using parameters previously described [69]. The primers used are described in Additional file 2. Results have been normalised to expression per Leydig cell as the transcripts measured here would be expected to be expressed specifically in steroidogenic/Leydig cells and Leydig numbers in all groups were increased by treatment with hCG.

For data presented in Fig. 5, RNA was extracted from either whole testes or cultured cells using RNeasy Mini kits (Qiagen Ltd., Manchester, UK). Random hexamer-primed cDNA was prepared using the VILO cDNA synthesis kit (Invitrogen, ThermoFisher Scientific, Paisley, UK) according to manufacturer’s instructions. The primers used for qPCR were designed using the online Roche Universal Probe Library (UPL) software (Roche Diagnostics Ltd., Burgess Hill, UK) and they are listed in Additional file 2. The qPCRs were carried out using an ABI Prism 7900 Sequence Detection System (ThermoFisher Scientific). Transcript levels per testis were normalised to luciferase using the ΔΔCt method [70] as above. Transcript levels in MLTC1 cells were normalised to an internal housekeeping gene assay for *Actb* (Roche).

### Statistical analysis

Data were analysed using t-tests, analysis of variance (ANOVA) (with differences between individual groups identified by Fisher’s post-hoc test or by t-test) or the Kruskal-Wallis test as appropriate. Where necessary, data were transformed using the Box-Cox method [71] to avoid heterogeneity of variance. Statistical analysis was carried out using Minitab v15 (Minitab Ltd., Coventry, UK).

## Additional files

Additional file 1: Raw data from figures and tables. Primary raw data from Figure S1-S5 and from Table S1. (XLS 73 kb)

Additional file 2: Primer sequences. Sequences of primers used for qPCR studies. (DOCX 13 kb)

## References

[1] Crawford PA, Sadovsky Y, Milbrandt J (1997). Nuclear receptor steroidogenic factor 1 directs embryonic stem cells toward the steroidogenic lineage. Mol Cell Biol.

[2] Gondo S, Yanase T, Okabe T, Tanaka T, Morinaga H, Nomura M (2004). SF-1/Ad4BP transforms primary long-term cultured bone marrow cells into ACTH-responsive steroidogenic cells. Genes Cells.

[3] Yang Y, Li Z, Wu X, Chen H, Xu W, Xiang Q (2017). Direct reprogramming of mouse fibroblasts toward Leydig-like cells by defined factors. Stem Cell Rep.

[4] Gondo S, Okabe T, Tanaka T, Morinaga H, Nomura M, Takayanagi R (2008). Adipose tissue-derived and bone marrow-derived mesenchymal cells develop into different lineage of steroidogenic cells by forced expression of steroidogenic factor 1. Endocrinology.

[5] Tanaka T, Gondo S, Okabe T, Ohe K, Shirohzu H, Morinaga H (2007). Steroidogenic factor 1/adrenal 4 binding protein transforms human bone marrow mesenchymal cells into steroidogenic cells. J Mol Endocrinol.

[6] Hatano O, Takakusu A, Nomura M, Morohashi K (1996). Identical origin of adrenal cortex and gonad revealed by expression profiles of Ad4BP/SF-1. Genes Cells.

[7] Bandiera R, Sacco S, Vidal VP, Chaboissier MC, Schedl A (2015). Steroidogenic organ development and homeostasis: a WT1-centric view. Mol Cell Endocrinol.

[8] Heikkila M, Peltoketo H, Leppaluoto J, Ilves M, Vuolteenaho O, Vainio S (2002). Wnt-4 deficiency alters mouse adrenal cortex function, reducing aldosterone production. Endocrinology.

[9] Val P, Jeays-Ward K, Swain A (2006). Identification of a novel population of adrenal-like cells in the mammalian testis. Dev Biol.

[10] Hu L, Monteiro A, Johnston H, King P, O'Shaughnessy PJ (2007). Expression of Cyp21a1 and Cyp11b1 in the fetal mouse testis. Reproduction.

[11] Smeets EE, Span PN, van Herwaarden AE, Wevers RA, Hermus AR, Sweep FC, Claahsen-van der Grinten HL (2015). Molecular characterization of testicular adrenal rest tumors in congenital adrenal hyperplasia: lesions with both adrenocortical and Leydig cell features. J Clin Endocrinol Metab.

[12] Engels M, Span PN, Mitchell RT, Heuvel JJTM, Marijnissen-van Zanten MA, van Herwaarden AE (2017). GATA transcription factors in testicular adrenal rest tumours. Endocr Connect.

[13] Baker PJ, Sha JA, McBride MW, Peng L, Payne AH, O'Shaughnessy PJ (1999). Expression of 3β-hydroxysteriod dehydrogenase type I and VI isoforms in the mouse testis during development. Eur J Biochem.

[14] Nef S, Shipman T, Parada LF (2000). A molecular basis for estrogen-induced cryptorchidism. Dev Biol.

[15] O'Shaughnessy PJ, Morris ID, Baker PJ (2008). Leydig cell re-generation and expression of cell signaling molecules in the germ cell-free testis. Reproduction.

[16] Kilcoyne KR, Smith LB, Atanassova N, MacPherson S, McKinnell C, van den Driesche S (2014). Fetal programming of adult Leydig cell function by androgenic effects on stem/progenitor cells. Proc Natl Acad Sci U S A.

[17] Ariyaratne HB, Mendis-Handagama SM, Hales DB, Mason JI (2000). Studies of the onset of Leydig precursor cell differentiation in the prepubertal rat testis. Biol Reprod.

[18] Haider SG, Servos G (1998). Ultracytochemistry of 3beta-hydroxysteroid dehydrogenase in Leydig cell precursors and vascular endothelial cells of the postnatal rat testis. Anat Embryol (Berl).

[19] Shima Y, Matsuzaki S, Miyabayashi K, Otake H, Baba T, Kato S (2015). Fetal Leydig cells persist as an androgen-independent subpopulation in the postnatal testis. Mol Endocrinol.

[20] O'Shaughnessy PJ, Johnston H, Willerton L, Baker PJ (2002). Failure of normal adult Leydig cell development in androgen-receptor-deficient mice. J Cell Sci.

[21] Murphy L, Jeffcoate IA, O'Shaughnessy PJ (1994). Abnormal Leydig cell development at puberty in the androgen-resistant *Tfm* mouse. Endocrinology.

[22] De Gendt K, Atanassova N, Tan KA, De Franca LR, Parreira GG, McKinnell C (2005). Development and function of the adult generation of Leydig cells in mice with Sertoli cell-selective (SCARKO) or total (ARKO) ablation of the androgen receptor. Endocrinology.

[23] O'Hara L, McInnes K, Simitsidellis I, Morgan S, Atanassova N, Slowikowska-Hilczer J (2015). Autocrine androgen action is essential for Leydig cell maturation and function, and protects against late-onset Leydig cell apoptosis in both mice and men. FASEB J.

[24] Naik SI, Young LS, Charlton HM, Clayton RN (1984). Pituitary gonadotropin-releasing hormone receptor regulation in mice. I: males. Endocrinology.

[25] Cattanach BM, Iddon CA, Charlton HM, Chiappa SA, Fink G (1977). Gonadtrophin releasing hormone deficiency in a mutant mouse with hypogonadism. Nature.

[26] O'Shaughnessy PJ, Verhoeven G, De Gendt K, Monteiro A, Abel MH (2010). Direct action through the sertoli cells is essential for androgen stimulation of spermatogenesis. Endocrinology.

[27] Scott IS, Charlton HM, Cox BS, Grocock CA, Sheffield JW, O'Shaughnessy PJ (1990). Effect of LH injections on testicular steroidogenesis, cholesterol side-chain cleavage P450 messenger RNA content and leydig cell morphology in hypogonadal mice. J Endocrinol.

[28] O'Shaughnessy PJ, Bennett MK, Scott IS, Charlton HM (1992). Effects of FSH on Leydig cell morphology and function in the hypogonadal mouse. J Endocrinol.

[29] Singh J, Oneill C, Handelsman DJ (1995). Induction of spermatogenesis by androgens in gonadotropin-deficient (hpg) mice. Endocrinology.

[30] O'Shaughnessy PJ, Baker PJ, Heikkila M, Vainio S, McMahon AP (2000). Localization of 17β-hydroxysteroid dehydrogenase/17-ketosteroid reductase isoform expression in the developing mouse testis - androstenedione is the major androgen secreted by fetal/neonatal leydig cells. Endocrinology.

[31] Song WC, Qian Y, Sun X, Negishi M (1997). Cellular localization and regulation of expression of testicular estrogen sulfotransferase. Endocrinology.

[32] Ivell R, Bathgate RA (2002). Reproductive biology of the relaxin-like factor (RLF/INSL3). Biol Reprod.

[33] Landreh L, Stukenborg JB, Soder O, Svechnikov K (2013). Phenotype and steroidogenic potential of PDGFRalpha-positive rat neonatal peritubular cells. Mol Cell Endocrinol.

[34] Welsh M, Saunders PT, Atanassova N, Sharpe RM, Smith LB (2009). Androgen action via testicular peritubular myoid cells is essential for male fertility. FASEB J.

[35] Xin HB, Deng KY, Rishniw M, Ji G, Kotlikoff MI (2002). Smooth muscle expression of Cre recombinase and eGFP in transgenic mice. Physiol Genomics.

[36] Lague E, Tremblay JJ (2008). Antagonistic effects of testosterone and the endocrine disruptor mono-(2-ethylhexyl) phthalate on INSL3 transcription in Leydig cells. Endocrinology.

[37] O'Shaughnessy PJ, Baker PJ, Johnston H (2006). The foetal Leydig cell - differentiation, function and regulation. Int J Androl.

[38] Claahsen-van der Grinten HL, Otten BJ, Stikkelbroeck MM, Sweep FC, Hermus AR (2009). Testicular adrenal rest tumours in congenital adrenal hyperplasia. Best Pract Res Clin Endocrinol Metab.

[39] Baker PJ, O'Shaughnessy PJ (2001). Role of gonadotrophins in regulating numbers of Leydig and Sertoli cells during fetal and postnatal development in mice. Reproduction.

[40] Zhang FP, Pakarainen T, Zhu F, Poutanen M, Huhtaniemi I (2004). Molecular characterization of postnatal development of testicular steroidogenesis in luteinizing hormone receptor knockout mice. Endocrinology.

[41] Ma X, Dong Y, Matzuk MM, Kumar TR (2004). Targeted disruption of luteinizing hormone beta-subunit leads to hypogonadism, defects in gonadal steroidogenesis, and infertility. Proc Natl Acad Sci U S A.

[42] Spaliviero JA, Jimenez M, Allan CM, Handelsman DJ (2004). Luteinizing hormone receptor-mediated effects on initiation of spermatogenesis in gonadotropin-deficient (hpg) mice are replicated by testosterone. Biol Reprod.

[43] O'Shaughnessy PJ (1991). Steroidogenic enzyme-activity in the hypogonadal (*hpg*) mouse testis and effect of treatment with luteinizing-hormone. J Steroid Biochem Mol Biol.

[44] Baker PJ, Johnston H, Abel MH, Charlton HM, O'Shaughnessy PJ (2003). Differentiation of adult-type Leydig cells occurs in gonadotrophin-deficient mice. Rep Biol Endocrinol.

[45] Davidoff MS, Middendorff R, Enikolopov G, Riethmacher D, Holstein AF, Muller D (2004). Progenitor cells of the testosterone-producing Leydig cells revealed. J Cell Biol.

[46] Smith LB, O'Shaughnessy PJ, Rebourcet D. Cell-specific ablation in the testis: what have we learned? Andrology. 2015;3:1035–1049. 10.1111/andr.12107 PMC4950036 26446427

[47] Goto M, Piper Hanley K, Marcos J, Wood PJ, Wright S, Postle AD (2006). In humans, early cortisol biosynthesis provides a mechanism to safeguard female sexual development. J Clin Invest.

[48] Johnston ZC, Bellingham M, Filis P, Soffientini U, Hough D, Bhattacharya S (2018). The human fetal adrenal produces cortisol but no detectable aldosterone throughout the second trimester. BMC Med.

[49] Yazawa T, Imamichi Y, Miyamoto K, Khan MR, Uwada J, Umezawa A, Taniguchi T (2016). Induction of steroidogenic cells from adult stem cells and pluripotent stem cells. Endocr J.

[50] Kuopio T, Pelliniemi LJ, Huhtaniemi I (1989). Rapid Leydig cell proliferation and luteinizing hormone receptor replenishment in the neonatal rat testis after a single injection of human chorionic gonadotropin. Biol Reprod.

[51] Dumontet T, Sahut-Barnola I, Septier A, Montanier N, Plotton I, Roucher-Boulez F (2018). PKA signaling drives reticularis differentiation and sexually dimorphic adrenal cortex renewal. JCI Insight.

[52] Padua MB, Jiang T, Morse DA, Fox SC, Hatch HM, Tevosian SG (2015). Combined loss of the GATA4 and GATA6 transcription factors in male mice disrupts testicular development and confers adrenal-like function in the testes. Endocrinology.

[53] Penny GM, Cochran RB, Pihlajoki M, Kyronlahti A, Schrade A, Hakkinen M (2017). Probing GATA factor function in mouse Leydig cells via testicular injection of adenoviral vectors. Reproduction.

[54] Xiao L, Feng Q, Zhang Z, Wang F, Lydon JP, Ittmann MM (2016). The essential role of GATA transcription factors in adult murine prostate. Oncotarget.

[55] Zhou Q, Nie R, Prins GS, Saunders PT, Katzenellenbogen BS, Hess RA (2002). Localization of androgen and estrogen receptors in adult male mouse reproductive tract. J Androl.

[56] Miyabayashi K, Shima Y, Inoue M, Sato T, Baba T, Ohkawa Y (2017). Alterations in fetal Leydig cell gene expression during fetal and adult development. Sex Dev.

[57] O'Shaughnessy PJ, Baker P, Sohnius U, Haavisto A-M, Charlton HM, Huhtaniemi I (1998). Fetal development of Leydig cell activity in the mouse is independent of pituitary gonadotroph function. Endocrinology.

[58] Zhang F-P, Poutanen M, Wilbertz J, Huhtaniemi I (2001). Normal prenatal but arrested postnatal sexual development of luteinizing hormone receptor knockout (LuRKO) mice. Mol Endocrinol.

[59] Rebourcet D, O'Shaughnessy PJ, Pitetti JL, Monteiro A, O'Hara L, Milne L (2014). Sertoli cells control peritubular myoid cell fate and support adult Leydig cell development in the prepubertal testis. Development.

[60] Lecureuil C, Fontaine I, Crepieux P, Guillou F (2002). Sertoli and granulosa cell-specific Cre recombinase activity in transgenic mice. Genesis.

[61] De Gendt K, Swinnen JV, Saunders PT, Schoonjans L, Dewerchin M, Devos A (2004). A Sertoli cell-selective knockout of the androgen receptor causes spermatogenic arrest in meiosis. Proc Natl Acad Sci U S A.

[62] O'Shaughnessy PJ, Monteiro A, Verhoeven G, De Gendt K, Abel MH (2009). Effect of FSH on testicular morphology and spermatogenesis in gonadotrophin-deficient hypogonadal (*hpg*) mice lacking androgen receptors. Reproduction.

[63] Mayhew TM (1992). A review of recent advances in stereology for quantifying neural structure. J Neurocytol.

[64] Wreford NG (1995). Theory and practice of stereological techniques applied to the estimation of cell number and nuclear volume of the testis. Microsc Res Tech.

[65] Vergouwen RPFA, Jacobs SGPM, Huiskamp R, Davids JAG, de Rooij DG (1991). Proliferative activity of gonocytes, sertoli cells and interstitial cells during testicular development in mice. J Reprod Fertil.

[66] O'Shaughnessy PJ, Murphy L (1993). Cytochrome P-450 17α-hydroxylase protein and mRNA in the testis of the testicular feminized (*Tfm*) mouse. J Mol Endocrinol.

[67] Baker PJ, O'Shaughnessy PJ (2001). Expression of prostaglandin D synthetase during development in the mouse testis. Reproduction.

[68] Johnston H, Baker PJ, Abel M, Charlton HM, Jackson G, Fleming L (2004). Regulation of Sertoli cell number and activity by follicle-stimulating hormone and androgen during postnatal development in the mouse. Endocrinology.

[69] Czechowski T, Bari RP, Stitt M, Scheible WR, Udvardi MK (2004). Real-time RT-PCR profiling of over 1400 Arabidopsis transcription factors: unprecedented sensitivity reveals novel root- and shoot-specific genes. Plant J.

[70] Livak KJ, Schmittgen TD (2001). Analysis of relative gene expression data using real-time quantitative PCR and the 2^(−ΔΔCT)^ method. Methods.

[71] Box G, Cox D (1964). An analysis of transformations. J Royal Stat Soc B.

