# Supplementary material for: Androgen receptor expression is required to ensure development of adult Leydig cells and to prevent development of steroidogenic cells with adrenal characteristics in the mouse testis
Source: BMC Dev Biol. 2019 Apr 17;19:8. doi: 10.1186/s12861-019-0189-5 (PMC6472051; doi:10.1186/s12861-019-0189-5)
Supplement: Supplementary file 2 — Primer sequences. Sequences of primers used for qPCR studies. (DOCX 13 kb) [file 12861_2019_189_MOESM2_ESM.docx]

Primers used for qPCR

A) SYBR primers

Gene Forward primer Reverse primer

Hsd3b1 cctcctaagggttaccctatatcataccagct gtctccttccaacactgtcaccttgg

Hsd3b6 gctccagactgggactgctgacac aatcctctggcccaaaaaccctc

Por gataccggagttcagcaagatccag ggtgggcatccttggacagc

Cyp11a1 cacagacgcatcaagcagcaaaa gcattgatgaaccgctgggc

Cyp17a1 tggtcccatctattctcttcgcctg aggcgacgccttttccttgg

Star cgtcggagctctctgcttggttc tcgtccccgttctcctgctg

Lhr caggaatttgccgaagaaagaacagaatt cagaagtcataatcgtaatcccagcca

Sult1e1 tgttgaaatgttcttggcaaggcc catcctccttgcatttttccacatca

Hsd17b3 atgggcagtgattaccggagca tacaatcttcacacagcttccagtggtc

Insl3 cacgcagcctgtggagaccc cgctggcgctgagaagcct

Mc2r attagtgacaaagccaaggagaggagca gggtggtgtttgccgttgacttac

Cyp11b1 tgcccttggaatcctggatagt ccattctggcccatttagcaa

Akr1b7 acatggctgccattctcagcttca ccaggttgattttagagagacgaaggaatct

Cyp21a1 accccatatgctaaatggaaagatggac gcatgcgctcacagaactcctg

Hao2 tcattgtttccaaccatggcgg agctccaagggctagtgccttca

Mrap cgctcaccagctatgagtattacctgga ccgaccaggacatgtagagcagga

B) UPL primers

UPL

Gene Forward primer Reverse primer probe

Ar ttatgaagcagggatgactctg gctgccagcattggagtt 12

Cyp11b1 ctggaaagtgtccatggtagc ccccaaaaagaacaaagtgg 47

Hsd17b3 aatatgtcacgatcggagctg gaagggatccggttcagaat 5

Insl3 aagaagccccatcatgacct tttatttagactttttgggacacagg 10

Mc2r catcttgccgagaaagatccta cctctccttggctttgtcac 96
